# Treating a friend to voter registration in a Divided America

**DOI:** 10.1371/journal.pone.0337176

**Published:** 2025-12-16

**Authors:** M. V. (Trey) Hood III, Seth C. McKee, Enrijeta Shino, Daniel A. Smith

**Affiliations:** 1 Department of Political Science, University of Georgia, Athens, Georgia, United States of America; 2 Department of Political Science, Oklahoma State University, Stillwater, Oklahoma, United States of America; 3 Department of Political Science, University of Alabama, Tuscaloosa, Alabama, United States of America; 4 Department of Political Science, University of Florida, Gainesville, Florida, United States of America; National Cheng Kung University, TAIWAN

## Abstract

As partisanship strengthens in the United States, unaffiliated voter registration is also increasing. We conducted an original survey experiment to understand voter registration choices among registered voters in Florida and North Carolina, two states with substantial shares of unaffiliated registrants and persistent partisan polarization, but with differing primary participation rules. Institutionally, Florida holds closed primaries where only those registered with a political party may participate in the respective primary for that party. North Carolina holds semi-closed primaries that allow those not registered with a political party (i.e., unaffiliated registrants) to vote in party primary elections. The results of our experiment demonstrate that institutional context matters. Specifically, informing respondents of primary participation rules shapes voter registration recommendations. Among registrants who identify as independents, exposure to Florida’s closed primary rule causes them to be more likely to recommend party registration, whereas awareness of the semi-closed North Carolina primary rule makes independents and partisans more likely to advise unaffiliated registration. However, in the absence of information explaining rules for primary participation, respondents are more likely to suggest that their friend register in alignment with their own political identity. In summary, our study provides valuable insights into how registration choices are conditioned by political identities and exposure to treatments that emphasize political conditions and primary participation rules.

## Introduction

Partisan sorting [1] and partisan polarization [2] are at contemporary highs, and yet Americans are registering with no party affiliation at record rates. Drawing on decades of national survey data, scholars have identified the relatively recent ascendance of *strong* partisans in the American electorate [3], but across the bulk of states that track party registration, the share of the electorate that registers independent has steadily increased since the late 1960s [4] (We do not display the data here, but from 2010 to 2022, we have documented the percentage of unaffiliated registrants in the 30 states with party registration. Compared to the percentage of their registered electorate in 2010, in 2022 unaffiliated registrants were a higher percentage of the registered electorate in twenty of these states (67%). (We suspect that while increasing partisan polarization among elites (officeholders and party activists) fuels mass-level partisanship, at the same time it likely has a repelling effect among large swaths of the electorate who respond by choosing not to register with a major party. We encourage other scholars to investigate this puzzle of rising partisanship alongside growing rates of unaffiliated voter registration.).

We examine voter registration choices in a manner that brings into focus the intersection of political affiliation, primary participation rules, and political polarization. To our knowledge, this is the first study to use an experimental approach to explain why individuals might support registering to vote with a major party or register unaffiliated. We find some evidence that a political polarization cue affects registration choices heterogeneously, whereas we consistently find political homophily and a state’s primary participation rules drive decisions about how one should register to vote.

We proceed by noting the strengthening partisanship amid rising unaffiliated voter registration in the United States. We then introduce our states of interest, Florida and North Carolina. Next, we offer our theory and hypotheses and describe our large-N survey experiment that emphasizes treatments regarding primary rules and partisan polarization to assess their possible effects on voter registration recommendations. Last, we present our findings, concluding with some thoughts on the important interplay between political identity, primary rules, partisan context, and voter registration choices.

## Strengthening partisanship, rising unaffiliated registration

The ongoing partisan sorting and partisan polarization within the American electorate [5] is fueled by this exaggerated condition found principally among the party in government [6] and party activists [7]. Americans appear to be polarizing in response to a deeply and increasingly divided partisan ruling class [8]. The impressive historical rebound in American partisanship (see Appendix F, S1–S3 Figs) after the palpable period of dealignment in the 1970s [9] has manifested from the late 1980s forward in greater voter loyalty and identification [3], nationalized voting behavior (e.g., a decline in ticket-splitting [10]), partisan polarization [2], and high turnout in the Trump years [11]. This development has concomitantly taken place alongside a rise in unaffiliated voter registration across the American states [4].

## Rules matter: Registration in Florida and North Carolina

What makes our research design compelling is that Florida and North Carolina have different registration rules allowing participation in party primaries, but both electorates show a substantial rise in voters not registered with a party (officially, No Party Affiliate (NPA) in Florida and Unaffiliated in North Carolina). For instance, from 2010 to 2022, according to the Florida Division of Elections, the share of NPA Florida registrants went from 19.5% to 27.5%. Likewise, over this same span of time, unaffiliated North Carolina registrants rose from 23.6% to 35.6%, according to the State Board of Elections. From a rational choice perspective [12], Florida’s registration rules should deter NPA registration because the state has closed primaries that allow only major party registered voters to participate in their party’s primary contests (If a general election in Florida is not contested, then all registered voters can participate in the only contested primary. In reality, this scenario almost never plays out because of a write-in candidate entering the general election.). Conversely, North Carolina’s semi-closed primary registration rules should foster unaffiliated registration, as unaffiliated voters may participate in either major party’s primary contest.

Though Florida and North Carolina show marked increases in unaffiliated registration, it makes sense that this rise is greater in the Tar Heel State due to its semi-closed primary rule. This said, at least some proportion of the growth in registered unaffiliated voters in both states is attributable to demographic changes, specifically increases in the types of people with a greater propensity to register unaffiliated. For instance, population growth tied to new residents, minorities, and younger voters correlate with higher unaffiliated registration, and the Florida and North Carolina electorates fit this profile (For North Carolina, see [25]; for Florida, see “Florida’s rapid rise of No Party Affiliation voters,” *Florida Trend*, October 22, 2022, https://floridatrend.com/article/34885/floridas-rapid-rise-of-no-party-affiliation-voters (last accessed May 10, 2024).) Also, both states have very high levels of partisan polarized rancor, especially among their elected officials who engage in cutthroat/hardball politics (e.g., partisan redistricting battles) (See, “Is North Carolina Going to Become Like Ron DeSantis’s Florida?” *Vanity Fair*, July 3, 2023, https://www.vanityfair.com/news/2023/06/north-carolina-governors-race-mark-robinson (last accessed May 10, 2024).).

We examine opinions toward voter registration in Florida and North Carolina because of these states’ different rules in a time of heightened partisan polarization among officeholders and their respective constituencies. However, knowing that rising unaffiliated registration is partly due to demographic changes within these states’ electorates, we intentionally elude this issue with a survey experiment on registered voters in both states. Underpinned by political homophily theory, our interest is identifying differences in voter registration recommendations (unaffiliated versus partisan) among validated registrants in both states who are independents (pure plus leaner) or partisans (strong plus weak). Relative to those in the control categories, randomly priming registered voters in both states with treatments emphasizing a primary registration rule (closed or semi-closed) or a partisan polarization cue, should capture an individual’s propensity to recommend a registration type (unaffiliated or partisan) for a hypothetical “friend” moving to either Florida or North Carolina. In short, we seek to explain how partisan polarization and primary rules may drive voter registration choices.

## Theory and hypotheses

Homophily, the alignment of individuals who are like-minded, is rampant in the political world [13]. The convergence of similar political preferences is a longstanding feature of the American electorate [14], showing up in numerous facets of daily life. Partisan sorting is a manifestation of political homophily, as an increasing number of voters line up their political beliefs/attitudes/ideological profiles with their affiliated party [15]. For instance, despite debate over the causal mechanisms leading to geographic sorting, Americans have displayed increasing partisan homogeneity in geographic space [16].

The crystallization of differences between the major parties [17] means that for many voters, identifying as an independent or partisan is a deliberate choice. Certainly, there is a notable share of “closet” partisans [18], similarly characterized as “undercover” partisans [19], but we know that in addition to self-identification, intensity of partisanship facilitates empirical analysis. Fortunately, differences in political behavior are consistently realized whether researchers classify independent leaners as independents *or* partisans. In our analysis of voter registration choices, we classify independent leaners as independents [20], but we obtain similar results if this category of respondent is coded as a partisan. In short, siding with [21], there is face validity to treating independent leaners as independents, because they identify this way on the initial party identification question.

Capturing common primary rules and the tenor of American politics, our survey experiment deploys two types of treatments to assess their effect on respondents’ voter registration recommendations: (1) information on the primary rules in Florida (closed primaries) or North Carolina (semi-closed primaries) and (2) a partisan polarization cue (The experiment is pre-registered in AsPredicted #138356 and is available https://aspredicted.org/N7G_BPP and in Appendix I.) Instead of asking respondents in Florida and North Carolina how *they* would register to vote (since they are already registered), we ask how they would recommend “a friend” register under two possible scenarios: (1) their friend is moving to Florida, *or* (2) their friend is moving to North Carolina. Our control condition provides no additional information beyond the hypothetical of a friend moving to either Florida or North Carolina, followed by asking respondents how their friend should register: unaffiliated or as a partisan. Under our design it is possible for a Florida respondent to be asked about a friend moving to North Carolina, and likewise for a North Carolina respondent to be asked about a friend moving to Florida. The randomized response options include: Register with the Democratic Party; Register with the Republican Party; Register as No Party Affiliation (FL language)/Unaffiliated (NC language); and Register with a third party. We also provide a “Don’t Know” response option (The survey questionnaire is found in Appendix J.).

Before asking respondents their voter registration recommendations, a group of randomly selected respondents received the primary rules treatment for one or the other state: “As you may know, in the state of Florida, registered Independents cannot participate in either major party’s (Democratic or Republican) primary election,” or “As you may know, in the state of North Carolina, registered unaffiliated voters can participate in either major party’s (Democratic or Republican) primary election.” Another randomly selected set of respondents received the partisan polarization cue: “As you may know, Democratic and Republican politicians are constantly fighting over hot-button issues in Florida [North Carolina]. This has been the state of affairs in Florida [North Carolina] for decades now, and likely will not change anytime soon.”

As such, respondents are placed in one of six possible conditions: (1) the control condition that provides no additional information beyond a friend moving to the respondent’s state; (2) no additional information beyond a friend moving to the other state; (3 and 4) addition of the partisan polarization cue for either Florida or North Carolina; and (5 and 6) the addition of primary rules—either the Florida closed primary or the North Carolina semi-closed primary. Because we randomly assign the partisan polarization cue or primary registration rules to respondents in both states, our experiment isolates the causal effects of these treatments on voter registration recommendations. Further, as per our expectations regarding homophily, political affiliation (independent or partisan) should condition registration choices varying according to exposure to the treatments.

Based on political homophily, we expect a respondent to recommend that their friend choose the same registration as their own (We acknowledge that our hypotheses represent a deviation from our preregistration, which stated a general expectation that respondents would be more likely to recommend registration with their aligned party. Upon further theoretical reflection, we recognized that this general prediction obscured important distinctions between partisans (who should already recommend their own party regardless of primary rules) and independents (who should shift toward partisan registration when informed about closed primaries). While not preregistered, we believe this theoretical refinement provides a more precise and theoretically sound approach to testing the effects of primary rules on registration recommendations.) Thus, we expect independents to recommend that their friend register unaffiliated, while partisans recommend their friend register as a partisan (We might expect Democrats to recommend Democratic registration and Republicans to recommend Republican registration, but we do not take this more specific approach as some partisans may recommend opposite party registration if living where the opposing party is electorally dominant [26].). Compared to no additional information, the partisan polarization cue should cause independents to be even *more* likely to recommend unaffiliated registration, because independents are more turned off by partisan polarization [22] (We note that this is a deviation from our preregistration plan, which did not specify separate hypotheses for partisans and independents.). Respondents receiving information about Florida’s closed primary rule should cause independents to be more likely to recommend their friend register as a partisan so they will not be excluded from participating in party primaries (Here we deviate from the preregistration plan by offering separate hypotheses for partisans and independents in the Florida closed primary context.). The North Carolina semi-closed primary rule should cause an increase in recommending independent registration because the rule gives unaffiliated voters the freedom to participate in either major party primary (This is again a deviation from the preregistration plan which does not contain separate hypotheses for partisans and independents. To clarify, North Carolina unaffiliated registrants can choose to participate in a Democratic *or* Republican primary exclusively, because these contests are necessarily limited to only candidates affiliated with that major party’s primary.).

With regard to partisans, versus those with no additional information, the partisan polarization cue should not affect voter registration recommendations because Democrats and Republicans are likely unfazed by this treatment. Likewise, while the Florida closed primary rule is restrictive, partisans should already be inclined to recommend that their friend register with a party. Hence, compared to the condition with no additional information, we expect Florida’s closed primary rule to have *a positive* effect on a partisan’s registration recommendation. Finally, the North Carolina semi-closed primary rule *should* incentivize partisans to recommend their friend register as unaffiliated, as the rule allows for participation in either major party’s primary (One can understand why partisans register unaffiliated in semi-closed primary states. For example, in a certain election the competition might occur in the opposing party’s primary (the partisan’s co-partisan candidate might be an unopposed incumbent seeking reelection or there may not be a co-partisan candidate running). One can also envision strategic behavior, like registering unaffiliated to vote for the “weakest” candidate in the opposing party’s primary [27].).

## Data and methods

We conducted two original survey experiments on Florida and North Carolina registered voters. The Florida survey was in the field July 18-28, 2023, and the North Carolina survey was in the field August 1-12, 2023. The Florida sample includes a random draw of 600,000 registrants from more than 15 million registered voters in the June 1, 2023 Florida voter file, of which 597,054 emails were valid (Monthly snapshots of the Florida voter file are publicly available, and they include emails. See Florida Division of Elections, “Voter Information as a Public Record,” https://dos.myflorida.com/elections/for-voters/voter-registration/voter-information-as-a-public-record/ (last accessed May 10, 2024).). The North Carolina sample was randomly drawn from over 7 million registered voters, and was purchased from the vendor Aristotle. The sample is a random draw of 610,271 registered voters, with 591,312 appended valid emails (Weekly snapshots of the North Carolina voter file are publicly available, but do not include emails. See North Carolina State Board of Elections, “Voter Registration Data,” https://www.ncsbe.gov/results-data/voter-registration-data (last accessed May 10, 2024).). Respondents received an email invitation that contained a link to the survey that was hosted on the Qualtrics platform. The response rate for the Florida survey was 1% and for the North Carolina survey it was 0.26% (We received recorded responses from 8,095 registered voters, with 6,016 completing the Florida survey. The calculated margin of error for the total sample is +/- 1.2 points at the 95% confidence level. For the North Carolina survey we received recorded responses from 1,941 registered voters, with 1,321 completing the survey. In this case the calculated margin of error for the total sample is +/- 2.5 points at the 95% confidence level. See Appendix H for our statement on APSA Council’s Principles and Guidance for Human Subjects Research.). Both web-based surveys received IRB approval and respondents were not compensated for their participation (The study was approved by IRB at the University of Alabama, the University of Florida, and the University of Georgia. Upon clicking on the survey link, a written consent script was displayed to respondents. Informed consent was recorded through the selection of a response option indicating such.).

**Fig 1:**
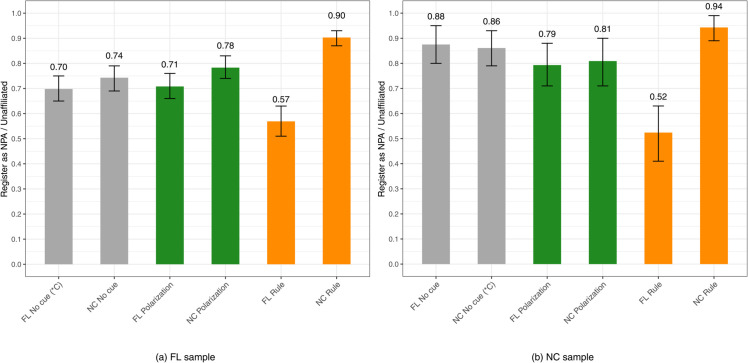
*Independents*’ voter registration recommendations: mean scores by treatment and control conditions (*C) signifies the control group. Gray bars represent control groups, green bars represent the polarization condition, and orange bars represent the institutional rule condition. Error bars represent 95% confidence intervals.

**Fig 2:**
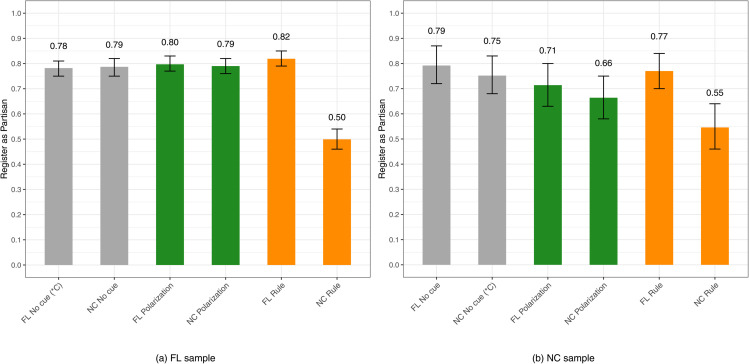
*Partisans*’ voter registration recommendations: mean scores by treatment and control conditions (*C) signifies the control group. Gray bars represent control groups, green bars represent the polarization condition, and orange bars represent the institutional rule condition. Error bars represent 95% confidence intervals.

We randomly assigned respondents from each state to the control or treatment groups. Respondents were not exposed to any other information about either state. Our empirical analysis has two parts. First, we assess the views of independents (pure plus leaner). For independents the variable is coded 1 if they advise their friend to register NPA/Unaffiliated, and 0 if they recommend their friend to register with a major party. Second, we examine the response patterns of partisans (strong plus weak). Here, the variable is coded 1 if partisans recommend their friend to register with a major party and 0 if they should register NPA/Unaffiliated (As indicated in the preregistration plan, the original dependent variable contained five categories which were later recoded into a binary form for ease of use for the analyses presented in Figs 1 and 2. Refer to Appendix A for a detailed description on variable coding.). The next section displays mean scores for the aforementioned six voter registration conditions for independents and partisans, respectively. In Appendices C-E, as a robustness check, we replicate our findings with linear and logistic regression and the results are consistent (We have a full model that controls for partisanship (pure independent to strong partisan). In the model limited to independents we include a dummy for independent leaners, and in the model limited to partisans we include a dummy for strong partisans.).

## Findings

In this section the results are confined to respondents who passed a simple attention check before we presented the survey experiment (The attention check, “To ensure you are a real person, please select the color red,” offered three randomized colors to choose from (blue, red, and white). We also estimate models including respondents who passed a manipulation check, shown in Appendix C, S2 Table, as well as all respondents (attention check) with a linear regression (Appendix D) and logistic regression (Appendix E). The findings are consistent across both specifications, suggesting our main conclusions are robust to the choice of filtering criteria.) In the accompanying figures, we show descriptive data (mean scores) for Florida on the left and North Carolina on the right. Reinforcing the reliability of the findings, consistently we find that the Florida sample appears more partisan in its registration recommendations. This comports with the state’s registration rules influencing political affiliation [20]. A state with a closed primary rule should make party identification more attractive than it would be in a state with a semi-closed primary rule. In line with this expectation, the 2022 Cooperative Election Study (CES) Florida sample had 66% partisans (strong plus weak) and 34% independents (pure plus leaner). By comparison, the 2022 CES North Carolina sample contained 60% partisans (strong plus weak) and 40% independents (pure plus leaner).

Fig 1 shows independents’ voter registration recommendations. In both state samples the NPA/Unaffiliated recommendation is typically higher in North Carolina. Primary rules are the main driver of voter registration recommendations. For independents in our Florida sample, exposure to Florida’s closed primary rule drops the NPA recommendation to .57, from .70 for the control group (Florida-no cue). For the North Carolina sample, informing independent respondents that unaffiliated registrants are not able to vote in Florida’s Democratic and Republican primary elections produces a recommendation for registering unaffiliated at .52, versus .86 for North Carolina independent respondents with no cue. In contrast, independents in both states exposed to North Carolina’s semi-closed primary rule recommend a friend to register NPA/Unaffiliated at a rate of .90 or higher (.94 for North Carolina independents).

Turning to independents in the Florida sample, the difference between those in the Florida no-cue category at .70, versus independents informed of North Carolina’s rule at .90, is statistically significant (see Appendix G S8 Table). In contrast to these primary rules treatments, the polarization cues do not exhibit notable movement in registration recommendations. For instance, among Florida independents, there are very modest differences in registration recommendations for the Florida no-cue condition (.70) versus the Florida polarization treatment (.71); and likewise for the North Carolina no-cue condition (.74) compared to the North Carolina polarization treatment (.78). Finally, among North Carolina independents, the no-cue conditions for Florida (.88) and North Carolina (.86), both exhibit higher NPA/Unaffiliated registration recommendations than the corresponding registration recommendations for independents exposed to the polarization cues for Florida (.79) or North Carolina (.81).

Next, in Fig 2 we examine partisans’ voter registration recommendations (see Appendix G S9 Table). The vertical axis is now set to recommending a friend to register with a major party (Democratic or Republican). Similar to independents recommending NPA/Unaffiliated registration, partisans recommend partisan registration. The only exception is in the case of partisans exposed to North Carolina’s semi-closed primary rule. For the Florida sample, the recommendation to register as a partisan plunges to .50 when partisan respondents are informed that NPAs/Unaffiliated registrants are able to vote in party primaries, versus .78 for Florida partisans receiving no cue. Also, the party registration recommendation is highest (.82) in the case of partisans receiving the Florida primary rule treatment. Among partisans in North Carolina, exposure to the state’s primary rule drops the recommendation to register with a major party to .55, as compared to .75 in the case of North Carolina partisans not receiving a cue (In other words, just like with the Florida sample, in the North Carolina sample, based on the 95% confidence intervals displayed in Fig 2, at this significance level, the clear differences materialize among partisans receiving the Florida closed primary rule versus partisans receiving the North Carolina semi-closed primary rule.).

## Conclusion

Over the years, scholars have examined voter registration patterns in the United States, but this is the first study to employ a large-N survey experiment to assess voter registration choices. We analyze the political behavior of respondents in two states with contentious partisan politics but different primary registration rules. In a polarized America, where many voters may not face closely fought general election contests, party primaries are increasingly determinative of who wins office. Our innovative experiment confirms a high degree of political homophily in registration recommendations. Specifically, the lion’s share of partisans recommend that their friend should register with a major party, whereas the bulk of independents advise their friend to register unaffiliated (We thank an anonymous reviewer who points out that there are likely other possible reasons why partisans recommend partisan registration while independents recommend unaffiliated registration. For instance, civic duty may possibly compel such like-minded behavior, but unfortunately our survey did not address such other possible motivations (like normative beliefs toward registering with a party or as unaffiliated). (Likewise, instead of homophily per se, it is possible that affective polarization drives co-partisan recommendations in the case of partisans, but this dynamic is not relevant to the behavior of independents, because they do not exhibit affective polarization.).

Political homophily in registration choices is either greatly reinforced or substantially undermined by exposure to different registration rules. Cued with Florida’s restrictive closed primary rule, independents shift in favor of recommending party registration. In contrast, exposure to North Carolina’s relatively expansive semi-closed primary rule causes independents and partisans in both states to move toward recommending NPA (Florida)/Unaffiliated (North Carolina) registration. The polarization cue, however, does not elicit a similar and anticipated effect on voter registration recommendations. Indeed, the only suggestive (though not significant) evidence for this treatment behaving in a fashion we might expect, manifests in the sample of North Carolina partisans.

Our findings are both important and illuminating. They demonstrate substantial variation in voter registration choices when the decision is essentially costless: how to advise a friend to register to vote upon moving to Florida or North Carolina. Of course, the registration choice is not costless with respect to participation and partisan politics. By deterring independent participation under the closed primary rule, more independents recommend major party registration, which fosters hidden partisanship in reverse (with actual independents registering as partisans). Similarly, by liberalizing primary participation, more partisans recommend NPA/Unaffiliated registration, which understates the true number of partisans on the voter rolls in semi-closed primary states.

Our experimental survey design allows us to assess voter registration choices apart from external influences. Besides North Dakota (the only state with no voter registration requirement), registering is costly [23] but highly variable [24]. Because certain demographic characteristics are more/less associated with registering with/without a party, this complicates investigations of voter registration choices. Also, there are registered voters affiliated with a party different from what their registration status shows, because they have not updated their registration to match this change. Therefore, the advantage of our survey experiment is that we sidestep these confounding factors to get a clearer picture of how registration rules and political conditions influence the electorally consequential decision of registering to vote.

## Supporting information

S1 Appendix Supplemental Information: Voter Registration Choices in a Polarized America. (PDF)

S1 Table Balance of covariates for Florida sample. (TEX)

S2 Table Balance of covariates for North Carolina sample. (TEX)

S3 Table Linear regression full models (attentive respondents). (TEX)

S4 Table Linear regression full models (respondents who passed the manipulation check). (TEX)

S5 Table Linear regression models for suggested party registration (all respondents). (TEX)

S6 Table Logistic regression models for suggested party registration (attentive respondents). (TEX)

S1 Fig Partisans and independents in America, ANES 1952–2020. (PDF)

S2 Fig Strength of partisanship in America, ANES 1952-2020. (PDF)

S3 Fig Strength of partisanship in America, CES 2006–2022. (PDF)

S7 Table Difference of means test-all respondents. (TEX)

S8 Table Difference of means test-independents. (TEX)

S9 Table Difference of means test-partisans. (TEX)

S1 Text Preregistration and questionnaire details. (PDF)

## References

[1] Levendusky M. The partisan sort. Chicago: University of Chicago Press; 2009.

[2] Campbell JE. Polarized: making sense of a Divided America. Princeton: Princeton University Press; 2016.

[3] Bartels LM. Partisanship and voting behavior 1952 -1996. American Journal of Political Science. 2000;44(1):35–50.

[4] McGhee E, Krimm D. Party registration and the geography of party polarization. Polity. 2009;41(3):345–67. doi: 10.1057/pol.2009.6

[5] Abramowitz AI. The polarized American electorate: the rise of partisan-ideological consistency and its consequences. Political Science Quarterly. 2022;137(4):645–74. doi: 10.1002/polq.13388

[6] Theriault SM. Party polarization in congress. Cambridge: Cambridge University Press; 2008.

[7] Layman GC, Carsey TM, Green JC, Herrera R, Cooperman R. Activists and conflict extension in American Party Politics. Am Polit Sci Rev. 2010;104(2):324–46. doi: 10.1017/s000305541000016x

[8] Hetherington MJ. Resurgent mass partisanship: the role of elite polarization. Am Polit Sci Rev. 2001;95(3):619–31. doi: 10.1017/s0003055401003045

[9] Abramson PR. Generational change and the decline of party identification in America: 1952 –1974. American Political Science Review. 1976;70(2):469–78.

[10] Jacobson GC. It’s nothing personal: the decline of the incumbency advantage in US House Elections. The Journal of Politics. 2015;77(3):861–73. doi: 10.1086/681670

[11] McDonald MP. From pandemic to insurrection: voting in the 2020 US presidential election. Boston: De Gruyter; 2022.

[12] Downs A. An economic theory of democracy. New York: HarperCollins; 1957.

[13] Huber GA, Malhotra N. Political homophily in social relationships: evidence from online dating behavior. The Journal of Politics. 2017;79(1):269–83. doi: 10.1086/687533

[14] Berelson BR, Lazarsfeld PF, McPhee WN. Voting: a study of opinion formation in a presidential campaign. Chicago: University of Chicago Press; 1954.

[15] Mason L. Uncivil agreement: how politics became our identity. Chicago: University of Chicago Press; 2018.

[16] Tam Cho WK, Gimpel JG, Hui IS. Voter migration and the geographic sorting of the American Electorate. Annals of the Association of American Geographers. 2013;103(4):856–70. doi: 10.1080/00045608.2012.720229

[17] Fiorina MP, Abrams SA, Pope JC. Polarization in the American Public: misconceptions and misreadings. The Journal of Politics. 2008;70(2):556–60. doi: 10.1017/s002238160808050x

[18] Keith BE, Magleby DB, Nelson CJ, Orr EA, Westlye MC. The myth of the independent voter. Berkeley: University of California Press. 1992.

[19] Klar S, Krupnikov Y. Independent politics. Cambridge: Cambridge University Press; 2016.

[20] Burden BC, Greene S. Party attachments and state election laws. Political Research Quarterly. 2000;53(1):63–76.

[21] Miller WE. Party identification, realignment, and party voting: back to the basics. Am Polit Sci Rev. 1991;85(2):557–68. doi: 10.2307/1963175

[22] Ansolabehere S, Iyengar S. Going negative: how attack ads shrink and polarize the electorate. New York: Free Press; 1995.

[23] Wolfinger RE, Rosenstone SJ. Who votes?. New Haven: Yale University Press; 1980.

[24] Seljan E, Lochner T, Webb A. The partisan costs of automatic voter registration. Electoral Studies. 2023;82:102591. doi: 10.1016/j.electstud.2023.102591

[25] Bitzer JM, Cooper CA, Manzo WR, Roberts S. Growing and distinct: the unaffiliated voter as unmoored voter. Social Science Quarterly. 2022;103(7):1587–601. doi: 10.1111/ssqu.13225

[26] Arrington TS, Grofman B. Party registration choices as a function of the geographic distribution of partisanship: a model of ‘hidden partisanship’ and an illustrative test. Political Geography. 1999;18(2):173–85. doi: 10.1016/s0962-6298(98)00071-7

[27] Cherry TL, Kroll S. Crashing the party: an experimental investigation of strategic voting in primary elections. Public Choice. 2003;114(3–4):387–420. doi: 10.1023/a:1022637002301

