# Supplementary material for: Treating a friend to voter registration in a Divided America
Source: PLoS One. 2025 Dec 16;20(12):e0337176. doi: 10.1371/journal.pone.0337176 (PMC12707647; doi:10.1371/journal.pone.0337176)
Supplement: S1 Appendix — (PDF) [file pone.0337176.s001.pdf]

S1 Appendix. Supplemental Information:

Voter Registration Choices in a Polarized America

Contents

A Variable Coding 1

B Balance of Covariates 3

C Linear Regression Full Models 4

D Robustness Check: Linear Regression, All Respondents 6

E Robustness Check: Logistic Regression, Attentive Respondents Only) 6

F Partisanship in America, ANES and CES Data 7

G Difference in Means 10

H Principles and Guidance for Human Subjects Research 11

I Pre-Registration 12

J Questionnaire 15

A Variable Coding

- **Dependent variable**:
  - ***Suggested registration party (all)*** is a dummy variable coded 1 if respondents would advise their friend to register NPA / Unaffiliated and 0 if they should register with a major or third party.
  - ***Suggested registration party (no third party)*** is a dummy variable coded 1 if the respondent would advise their friend to register as NPA / Unaffiliated and 0 if should register as a major party affiliate.

    Suggested registration party (major party) is a dummy variable coded 1 if the respondent would advise their friend to register with major party and 0 if they should register as NPA / Unaffiliated.
- **Control group**:
  - ***C FL/NC no cue (about the state in which the respondent resides) (text)***: A friend of yours is moving to (respondent's home-state) Florida/North Carolina. They are interested in registering to vote. How would you advise your friend to register to vote in Florida/North Carolina?
- **Treatment groups**:
  - ***T1: FL/NC no cue (about the state in which the respondent does not reside) (text)***: A friend of yours is moving to Florida/North Carolina. They are interested in registering to vote. How would you advise your friend to register to vote in Florida/North Carolina?
  - ***T2: FL Partisan Polarization (text)***: A friend of yours is moving to Florida. They are interested in registering to vote. As you may know, as in North Carolina, Democratic and Republican politicians in Florida are constantly fighting over hot-button issues. This has been the state of affairs in Florida for decades now, and likely will not change anytime soon. How would you advise your friend to register to vote in Florida?
  - ***T3: NC Partisan Polarization (text)***: A friend of yours is moving to North Carolina. They are interested in registering to vote. As you may know, Democratic and Republican politicians are constantly fighting over hot-button issues in North Carolina. This has been the state of affairs in North Carolina for decades now, and likely will not change anytime soon. How would you advise your friend to register to vote in North Carolina?
  - ***T4: FL Closed Primary (text)***: A friend of yours is moving to Florida. They are interested in registering to vote. As you may know, in the state of Florida, registered Independents cannot participate in either major party's (Democratic or Republican) primary election. How would you advise your friend to register to vote in Florida?
  - ***T5: NC Semi-Closed Primary (text)***: A friend of yours is moving to North Carolina. They are interested in registering to vote. As you may know, in the state of North Carolina, registered Independents can participate in either major party's (Democratic or Republican) primary election. How would you advise your friend to register to vote in North Carolina?

- **Control variables**:
  - ***Partisanship (pure → strong)*** is a categorical variable coded as: 1 = pure independent, 2 = independent leaner, 3 = weak partisan, 4 = strong partisan.
  - ***Independent leaner*** is a dummy variable coded 1 if independent respondent self-identified as leaning toward a major party and 0 for pure independents.
  - ***Strong partisan*** is a dummy variable coded 1 if partisan respondent self-identified as a strong partisan and 0 if self-identified as a weak partisan.
  - ***Vote in primary important*** is a categorical variable for how important it is to vote in primary election varying from 1 (not important) to 4 (very important).
  - ***Ideology*** is a seven point variable varying from: 1 = strong conservative to 7 = strong liberal.
  - ***Surrounded by like-minded*** is a dummy variable coded 1 if respondent agrees that they prefer to surround themselves with politically like minded people and 0 if they disagree.
  - ***Age*** is a continuous variable varying from 18 to 100 years old.
  - ***Female*** is a dummy variable coded 1 for female and 0 for male.
  - ***White*** is a dummy coded 1 for White non-Hispanic and 0 other.
  - ***Education*** is a categorical variable coded 1 if respondent has high school degree or less, 2 some college, 3 college degree, and 4 graduate degree.
  - ***Political awareness*** is a categorical variable varying from 1 if respondents hardly at all follows news about politics to 4 if they follow news most of the time.
  - ***Political engagement scale*** is a scale composed of six items on whether the respondents has 1) attended a local political meetings (such as school board or city council), 2) put up a political sign (such as a lawn sign or bumper sticker), 3) worked for a candidate or campaign, 4) attended a political protest, march or demonstration, 5) contacted a public official, 6) donated money to a candidate, campaign, or political organization. The scale varies from 0 (no engagement) to 5 (high engagement) (due to the low number of cases on group 6, we merged it with group 5). Scale's Cronbach alpha is 0.6.
  - ***Moved to Florida*** is a dummy variable coded 1 if respondent moved to Florida from another state and 0 if they have not.
  - ***Moved to North Carolina*** is a dummy variable coded 1 if respondent moved to North Carolina from another state and 0 if they have not.

B Balance of Covariates

Table S1: Balance of covariates for Florida sample

	Control			No information NC			Election rule cue NC			Election rule cue FL			Polarization cue NC			Polarization cue FL		
	mean	*sd*	*n*	*mean*	*sd*	*n*	*mean*	*sd*	*n*	*mean*	*sd*	*n*	*mean*	*sd*	*n*	*mean*	*sd*	*n*
Party registration suggestion (DV) [1 → 4]	1.94	0.84	1005	1.95	0.86	973	1.62	0.89	1061	2.09	0.83	979	1.91	0.85	985	1.97	0.88	1013
Political engagement scale [0 → 5]	1.01	1.21	987	1.06	1.22	948	1.02	1.19	1049	1.09	1.28	964	1.08	1.27	976	1.08	1.28	996
Vote in primaries important [1 → 4]	3.75	0.53	988	3.69	0.56	959	3.72	0.56	1040	3.73	0.55	968	3.76	0.50	964	3.73	0.51	991
Partisanship [1 → 4]	3.08	1.08	988	3.11	1.07	953	3.01	1.07	997	3.06	1.08	971	3.19	1.02	960	3.04	1.09	1042
Independent leaner [0 → 1]	0.70	0.46	334	0.71	0.45	313	0.73	0.45	370	0.71	0.45	338	0.75	0.44	281	0.71	0.45	381
Strong partisan [0 → 1]	0.79	0.41	654	0.80	0.40	640	0.76	0.43	627	0.78	0.41	633	0.78	0.41	679	0.80	0.40	661
Political awareness [1 → 4]	3.72	0.59	1004	3.73	0.57	973	3.75	0.58	1060	3.69	0.60	979	3.75	0.54	984	3.71	0.61	1010
Surrounded by likeminded people [0 → 1]	0.53	0.50	931	0.59	0.49	879	0.58	0.49	970	0.57	0.50	884	0.57	0.50	893	0.55	0.50	923
Ideology [1 → 7]	3.91	0.85	1004	3.86	1.89	971	3.91	1.84	1059	3.93	1.83	978	3.88	1.84	983	3.99	1.82	1011
Move to FL [0 → 1]	0.77	0.42	1001	0.75	0.43	968	0.77	0.42	1056	0.77	0.42	975	0.77	0.42	980	0.77	0.42	1008
Age [19 → 100]	62.09	14.85	981	61.09	15.55	966	61.21	15.14	1048	61.81	14.91	972	61.73	15.15	973	61.48	15.73	1006
Female [0 → 1]	0.58	0.49	990	0.59	0.49	973	0.58	0.49	1052	0.58	0.49	972	0.58	0.49	973	0.56	0.50	1005
White non-Hispanic [0 → 1]	0.81	0.39	1001	0.76	0.42	970	0.77	0.42	1008	0.78	0.42	977	0.79	0.41	974	0.76	0.43	1057
Education [1 → 4]	3.03	0.97	1003	3.02	0.99	972	3.08	0.95	1060	3.06	0.97	977	3.09	0.94	985	3.01	0.99	1012

Notes: Table entries are unweighted descriptive statistics for control and treatment group. Variable range shown in brackets.

Table S2: Balance of covariates for North Carolina sample

	Control			No information FL			Election rule cue NC			Election rule cue FL			Polarization cue NC			Polarization cue FL		
	mean	*sd*	*n*	*mean*	*sd*	*n*	*mean*	*sd*	*n*	*mean*	*sd*	*n*	*mean*	*sd*	*n*	*mean*	*sd*	*n*
Party registration suggestion (DV) [1 → 4]	1.74	0.84	239	1.76	0.81	205	1.78	0.88	190	1.71	0.82	204	1.51	0.77	214	2.01	0.84	232
Political engagement scale [0 → 5]	1.06	1.28	234	1.09	1.24	201	1.16	1.25	187	1.15	1.35	201	0.96	1.18	208	1.04	1.23	227
Vote in primaries important [1 → 4]	3.75	0.51	238	3.74	0.46	202	3.77	0.43	188	3.73	0.50	204	3.79	0.45	212	3.79	0.44	227
Partisanship [1 → 4]	2.89	1.15	235	3.02	1.08	203	3.06	1.1	187	2.87	1.14	201	2.96	1.09	209	3.02	1.10	226
Independent leaner [0 → 1]	0.68	0.47	102	0.79	0.41	81	0.74	0.44	70	0.69	0.47	89	0.78	0.42	89	0.72	0.45	86
Strong partisan [0 → 1]	0.82	0.39	133	0.84	0.37	122	0.85	0.35	117	0.8	0.4	112	0.83	0.37	120	0.82	0.38	140
Political awareness [1 → 4]	3.74	0.59	239	3.78	0.52	205	3.73	0.57	190	3.79	0.52	204	3.78	0.52	214	3.78	0.53	232
Surrounded by likeminded people [0 → 1]	0.52	0.50	214	0.62	0.49	186	0.58	0.49	179	0.60	0.49	188	0.57	0.50	194	0.54	0.50	213
Ideology [1 → 7]	3.74	1.89	238	3.56	1.90	202	3.91	1.97	189	3.62	1.79	202	3.66	1.78	211	3.72	1.84	227
Move to NC [0 → 1]	0.72	0.45	231	0.67	0.47	201	0.70	0.46	186	0.74	0.44	197	0.68	0.47	207	0.73	0.45	223
Age [21 → 100]	64.54	12.08	228	65.14	13.11	200	63.20	13.20	179	67.34	11.85	194	67.10	11.67	205	65.26	13.09	220
Female [0 → 1]	0.58	0.49	229	0.55	0.50	202	0.57	0.50	183	0.65	0.48	197	0.58	0.50	206	0.62	0.49	223
White non-Hispanic [0 → 1]	0.83	0.37	193	0.88	0.33	175	0.79	0.41	145	0.88	0.33	174	0.87	0.34	180	0.81	0.39	182
Education [1 → 4]	2.94	1.04	232	2.82	0.96	202	2.92	0.98	185	2.98	1.00	197	2.89	0.99	207	2.92	1.01	226

Notes: Table entries are unweighted descriptive statistics for control and treatment group. Variable range shown in brackets.

C Linear Regression Full Models

Table S3: Linear regression full models for suggested party registration: Attentive respondents only

	**DV**: NPA = 1		**DV**: NPA = 1		**DV**: Major party = 1	
	FL	NC	FL	NC	FL	NC
	(Full sample)	(Full sample)	(Independents only)	(Independents only)	(Partisans only)	(Partisans only)
FL no cue	0.024		0.088*		0.007	
	(0.020)		(0.037)		(0.024)	
NC no cue		0.024		0.103		0.018
		(0.043)		(0.066)		(0.056)
FL Partisan Polarization	0.001	0.033	0.055	−0.011	0.027	−0.070
	(0.020)	(0.044)	(0.035)	(0.064)	(0.024)	(0.058)
NC Partisan Polarization	0.046*	0.075	0.142***	−0.013	0.006	−0.135*
	(0.020)	(0.044)	(0.036)	(0.067)	(0.024)	(0.057)
FL Closed Primary	−0.052**	−0.115**	−0.094*	−0.278***	0.045	0.014
	(0.020)	(0.042)	(0.039)	(0.066)	(0.024)	(0.055)
NC Semi-Closed Primary	0.258***	0.199***	0.252***	0.133*	−0.287***	−0.228***
	(0.020)	(0.043)	(0.035)	(0.063)	(0.024)	(0.058)
Partisanship (pure→strong)	−0.185***	−0.205***				
	(0.006)	(0.013)				
Independent leaner			−0.137***	−0.114*		
			(0.025)	(0.048)		
Strong partisan					0.162***	0.288***
					(0.019)	(0.047)
Vote in primary important	−0.094***	0.003	−0.097***	0.013	0.094***	0.009
	(0.012)	(0.029)	(0.016)	(0.040)	(0.018)	(0.040)
Ideology	−0.028***	−0.038*	−0.023	−0.065**	0.033***	0.011
	(0.007)	(0.016)	(0.013)	(0.025)	(0.009)	(0.021)
Age	0.000	−0.000	0.001	0.002	0.000	0.002
	(0.000)	(0.001)	(0.001)	(0.002)	(0.001)	(0.001)
Female	−0.030*	−0.051	0.004	−0.052	0.044**	0.063
	(0.012)	(0.026)	(0.023)	(0.040)	(0.014)	(0.034)
White	−0.004	−0.059	−0.006	−0.094	0.002	0.036
	(0.015)	(0.037)	(0.026)	(0.057)	(0.018)	(0.048)
Education	−0.004	0.012	0.006	−0.020	0.010	−0.033
	(0.006)	(0.013)	(0.011)	(0.020)	(0.007)	(0.017)
Surrounded by like-minded	−0.087***	−0.118***	−0.069**	−0.094*	0.101***	0.148***
	(0.012)	(0.027)	(0.022)	(0.040)	(0.015)	(0.036)
Political awareness	−0.014	−0.024	−0.011	−0.002	0.023	0.061
	(0.011)	(0.027)	(0.018)	(0.040)	(0.015)	(0.035)
Political engagement scale	−0.019***	−0.032**	−0.037***	−0.040*	0.012*	0.026*
	(0.005)	(0.011)	(0.009)	(0.017)	(0.005)	(0.013)
Moved to FL	0.067***		0.031		−0.074***	
	(0.015)		(0.028)		(0.017)	
Moved to NC		−0.010		−0.015		0.008
		(0.029)		(0.045)		(0.037)
(Intercept)	1.438***	1.410***	1.171***	1.140***	0.031	0.028
	(0.060)	(0.149)	(0.090)	(0.209)	(0.087)	(0.209)
R^2	0.303	0.334	0.136	0.196	0.154	0.178
Adj. R^2	0.301	0.323	0.127	0.162	0.150	0.157
Num. obs.	4,980	1,049	1,561	402	3,351	640

Note: Linear regression model for attentive respondents. The control group for Florida sample received no cue for North Carolina, and the control group for North Carolina sample received no cue about Florida.

Table S4: Linear regression full models for suggested party registration: Respondents who passed the manipulation check only

	**DV**: NPA = 1		**DV**: NPA = 1		**DV**: Major party = 1	
	FL	NC	FL	NC	FL	NC
	(Full sample)	(Full sample)	(Independents only)	(Independents only)	(Partisans only)	(Partisans only)
FL no cue	0.028		0.093*		0.006	
	(0.021)		(0.038)		(0.025)	
NC no cue		0.023		0.126		0.023
		(0.046)		(0.069)		(0.060)
FL Partisan Polarization	0.005	0.030	0.070	−0.024	0.027	−0.078
	(0.021)	(0.045)	(0.037)	(0.064)	(0.025)	(0.061)
NC Partisan Polarization	0.049*	0.063	0.145***	−0.036	0.003	−0.136*
	(0.021)	(0.046)	(0.038)	(0.070)	(0.025)	(0.060)
FL Closed Primary	−0.053*	−0.117**	−0.085*	−0.274***	0.050*	0.015
	(0.021)	(0.044)	(0.041)	(0.069)	(0.024)	(0.058)
NC Semi-Closed Primary	0.258***	0.189***	0.266***	0.138*	−0.280***	−0.221***
	(0.021)	(0.046)	(0.037)	(0.066)	(0.025)	(0.061)
Partisanship (pure→strong)	−0.186***	−0.204***				
	(0.007)	(0.014)				
Independent leaner			−0.143***	−0.131**		
			(0.026)	(0.051)		
Strong partisan					0.165***	0.296***
					(0.020)	(0.051)
Vote in primary important	−0.089***	−0.010	−0.093***	−0.019	0.086***	0.012
	(0.012)	(0.031)	(0.017)	(0.043)	(0.018)	(0.042)
Ideology	−0.030***	−0.043*	−0.020	−0.063*	0.038***	0.019
	(0.008)	(0.017)	(0.014)	(0.026)	(0.009)	(0.022)
Age	0.000	−0.000	0.001	0.003	0.000	0.002
	(0.000)	(0.001)	(0.001)	(0.002)	(0.001)	(0.002)
Female	−0.029*	−0.053	0.003	−0.069	0.042**	0.057
	(0.013)	(0.028)	(0.024)	(0.042)	(0.015)	(0.036)
White	−0.002	−0.031	0.016	−0.072	0.008	0.004
	(0.016)	(0.039)	(0.027)	(0.060)	(0.019)	(0.050)
Education	−0.003	0.015	0.004	−0.011	0.008	−0.031
	(0.006)	(0.014)	(0.012)	(0.021)	(0.008)	(0.018)
Surrounded by like-minded	−0.087***	−0.123***	−0.077**	−0.104*	0.097***	0.150***
	(0.013)	(0.028)	(0.023)	(0.042)	(0.016)	(0.038)
Political awareness	−0.017	−0.010	−0.015	0.020	0.025	0.050
	(0.012)	(0.027)	(0.019)	(0.040)	(0.015)	(0.036)
Political engagement scale	−0.019***	−0.032**	−0.037***	−0.032	0.012*	0.029*
	(0.005)	(0.011)	(0.010)	(0.018)	(0.006)	(0.014)
Moved to FL	0.062***		0.028		−0.065***	
	(0.015)		(0.029)		(0.018)	
Moved to NC		0.005		0.001		−0.012
		(0.031)		(0.048)		(0.039)
(Intercept)	1.428***	1.399***	1.146***	1.091***	0.037	0.037
	(0.062)	(0.155)	(0.094)	(0.219)	(0.090)	(0.216)
R^2	0.305	0.338	0.140	0.208	0.152	0.177
Adj. R^2	0.303	0.326	0.131	0.171	0.148	0.154
Num. obs.	4,551	950	1,416	361	3,071	583

Note: Linear regression model for respondents who passed the manipulation check. The control group for Florida sample received no cue for North Carolina, and the control group for North Carolina sample received no cue about Florida.

D Robustness Check: Linear Regression, All Respondents

Table S5: Linear regression models for suggested party registration

	**DV**: NPA = 1		**DV**: NPA = 1		**DV**: Major party = 1	
	FL	NC	FL	NC	FL	NC
	(Full sample)	(Full sample)	(Independents only)	(Independents only)	(Partisans only)	(Partisans only)
(Intercept)	0.377***	0.508***	0.697***	0.860***	0.778***	0.752***
	(0.015)	(0.032)	(0.024)	(0.038)	(0.017)	(0.039)
FL no cue	0.007		0.051		0.009	
	(0.022)		(0.034)		(0.024)	
NC no cue		−0.040		0.015		0.048
		(0.047)		(0.057)		(0.056)
FL Partisan Polarization	0.004	0.004	0.009	−0.067	0.019	−0.038
	(0.022)	(0.047)	(0.033)	(0.055)	(0.024)	(0.057)
NC Partisan Polarization	0.030	−0.003	0.083*	−0.063	0.011	−0.088
	(0.022)	(0.048)	(0.033)	(0.059)	(0.024)	(0.057)
FL Closed Primary	−0.087***	−0.177***	−0.128***	−0.336***	0.038	0.020
	(0.022)	(0.045)	(0.035)	(0.056)	(0.023)	(0.054)
NC Semi-Closed Primary	0.246***	0.155***	0.209***	0.084	−0.279***	−0.202***
	(0.021)	(0.046)	(0.033)	(0.055)	(0.024)	(0.056)
R^2	0.044	0.039	0.052	0.111	0.064	0.033
Adj. R^2	0.044	0.035	0.050	0.102	0.063	0.027
Num. obs.	5,933	1,280	1,933	507	3,809	741

Note: Linear regression model for all respondents (attentive and non attentive). The control group for Florida sample received no cue for North Carolina, and the control group for North Carolina sample received no cue about Florida.

E Robustness Check: Logistic Regression, Attentive Respondents Only)

Table S6: Logistic regression models for suggested party registration

	**DV**: NPA = 1		**DV**: NPA = 1		**DV**: Major party = 1	
	FL	NC	FL	NC	FL	NC
	(Full sample)	(Full sample)	(Independents only)	(Independents only)	(Partisans only)	(Partisans only)
(Intercept)	−0.503***	0.034	0.826***	1.815***	1.260***	1.109***
	(0.066)	(0.130)	(0.123)	(0.288)	(0.096)	(0.201)
FL no cue	0.027		0.246		0.042	
	(0.094)		(0.181)		(0.137)	
NC no cue		−0.153		0.131		0.267
		(0.192)		(0.444)		(0.304)
FL Partisan Polarization	0.010	0.007	0.032	−0.474	0.104	−0.218
	(0.093)	(0.193)	(0.170)	(0.397)	(0.139)	(0.290)
NC Partisan Polarization	0.123	−0.012	0.429*	−0.391	0.065	−0.428
	(0.093)	(0.195)	(0.181)	(0.422)	(0.138)	(0.281)
FL Closed Primary	−0.395***	−0.720***	−0.523**	−1.718***	0.242	0.089
	(0.098)	(0.191)	(0.176)	(0.363)	(0.139)	(0.285)
NC Semi-Closed Primary	0.993***	0.645***	1.422***	0.982	−1.254***	−0.923***
	(0.092)	(0.195)	(0.218)	(0.543)	(0.125)	(0.272)
Log Likelihood	−3839.190	−852.102	−1034.527	−221.330	−2028.962	−431.438
Num. obs.	5,855	1,265	1,900	498	3,766	735

Note: Logistic regression model for attentive respondents only. The control group for Florida sample received no cue for North Carolina, and the control group for North Carolina sample received no cue about Florida.

F Partisanship in America, ANES and CES Data

Fig S1. Partisans and Independents in America, 1952-2020

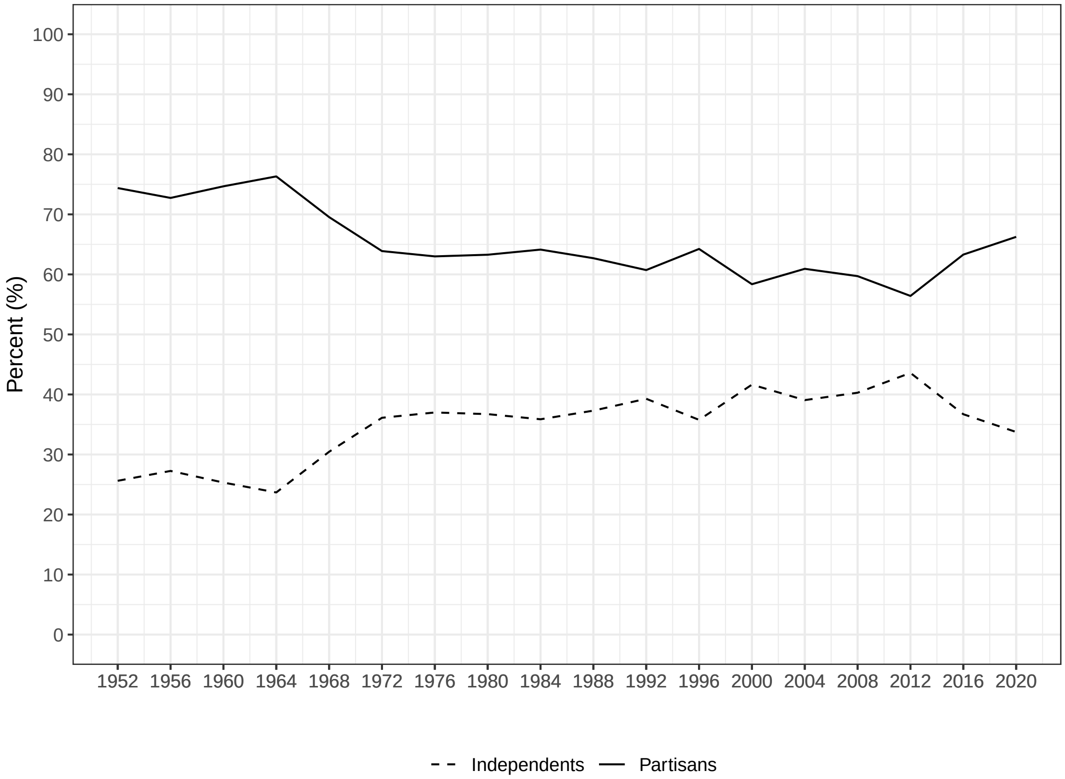

Note: ANES data compiled by the authors. Over the entirety of the American National Election Studies (ANES) time series in presidential years (1952-2020), the percentage of partisans (strong plus weak) in 2020 reached its highest level (66.3%) since 1968 (69.5%). Conversely, the share of independents (pure plus leaner) in 2020 (33.7%) was at its lowest point since 1968 (30.5%). American National Election Studies, "ANES 2020 Time Series Study Full Release [dataset and documentation]," February 10, 2022 version, available www.electionstudies.org (last accessed May 11, 2024).

Fig S2. Strength of Partisanship in America, ANES 1952-2020

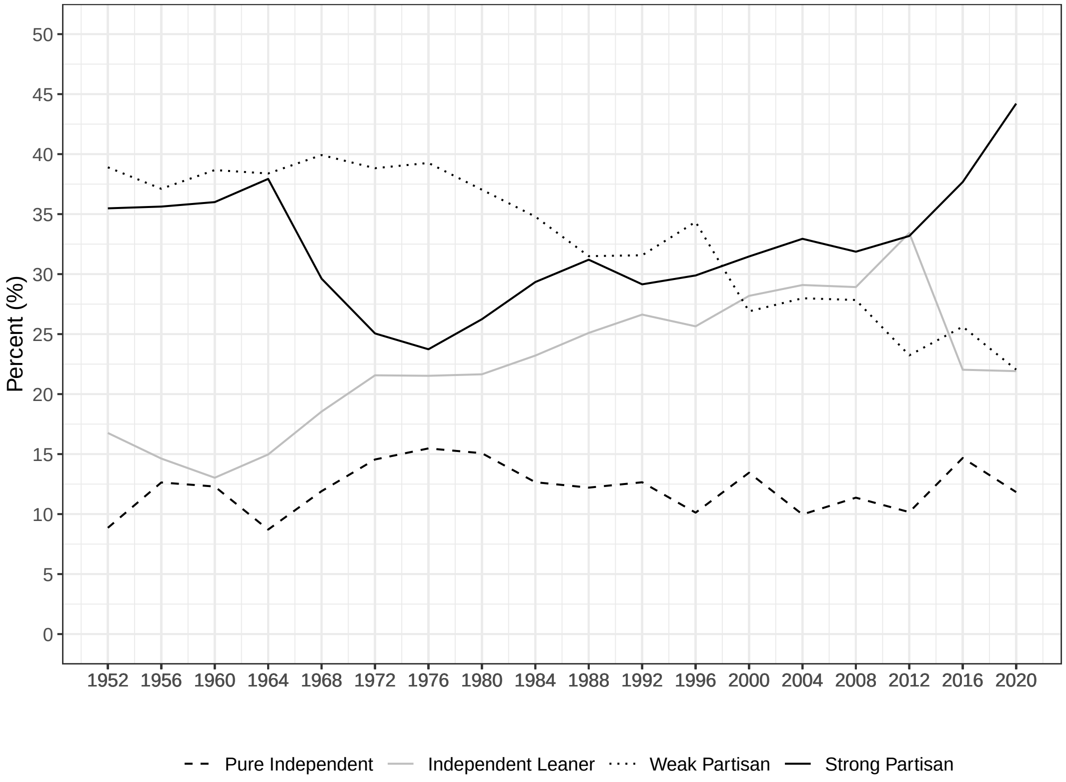

Note: ANES data compiled by the authors. When parsing the ANES data further, according to strength of partisanship (pure independent, independent leaner, weak partisan, and strong partisan), 2020 is the apex for any category, with strong partisans the plurality group at 44.2%. In 2012, the share of partisans in the ANES time series was at an all-time low (56.4%), but it rebounded in the next two presidential elections (almost a 10-percentage point increase). Even during the stable partisan period of the 1950s, and into the mid-1960s when the share of partisans (strong plus weak) peaked at 76.3% in 1964, weak partisans *always* outnumbered strong partisans in the ANES time series. In fact, it was not until the 2000 election when strong partisans in the ANES surpassed weak partisans, persisting ever since. The greatest disparity favoring weak partisans (39.3%) over strong partisans (23.7%) was 15.6 points in 1976 (around the height of dealignment). The greatest difference favoring strong partisans (44.2%) over weak partisans (22.0%) is 22.2 points in 2020, which is the only time one political affiliation group (pure independent, independent leaner, weak partisan, strong partisan) has ever accounted for over 40%. American National Election Studies, "ANES 2020 Time Series Study Full Release [dataset and documentation]," February 10, 2022 version, available www.electionstudies.org (last accessed May 11, 2024).

Fig S3. Strength of Partisanship in America, CES 2006-2022

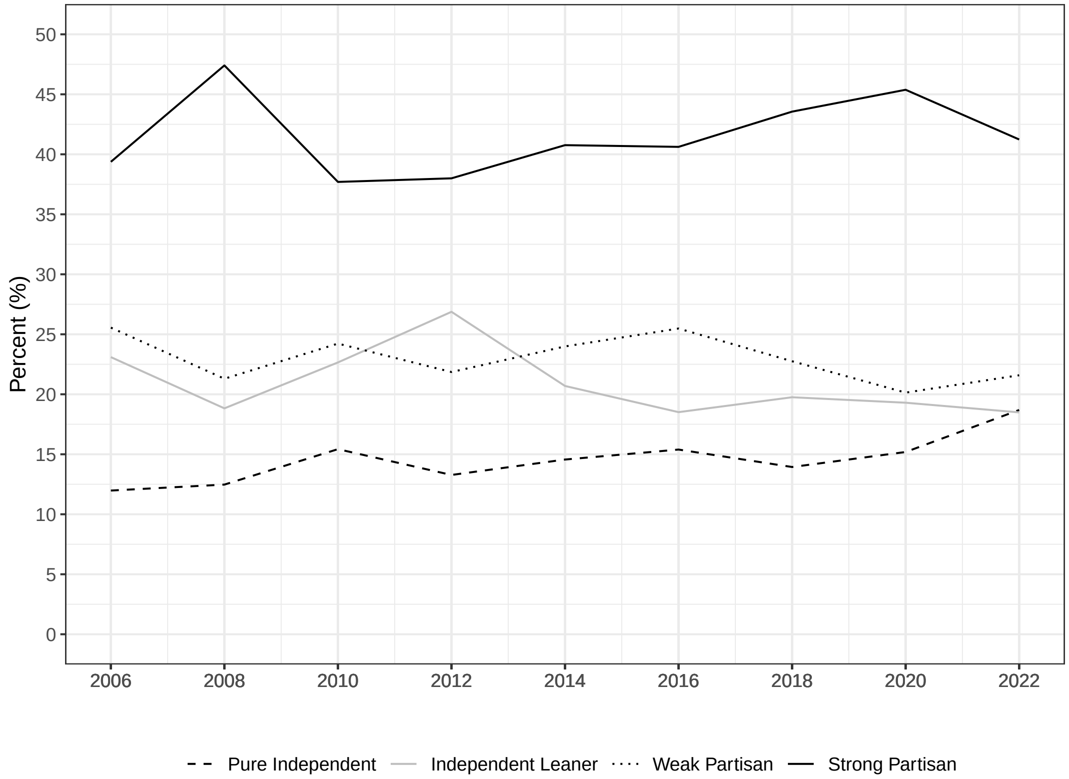

Note: CES data compiled by the authors. According to the 2022 Cooperative Election Study (CES), partisans (strong plus weak) account for 62.8% of respondents, with independents (pure plus leaner) making up 37.2%. At 41.2%, strong partisans in the 2022 CES are by far the largest group. The distribution for the other three categories in the 2022 CES are: 21.6% weak partisans, 18.5% independent leaners, and 18.7% pure independents. Interestingly, and in contrast to the ANES, for the entire CES time series from 2006 to 2022 (including midterm elections), strong partisans have always been the plurality group, and their share of the electorate has exceeded 40% in every election since 2014. Similar to the ANES, the 2012 CES is also the low-point in the share of partisans (strong plus weak = 59.9%) versus independents (pure plus leaner = 40.1%). Brian Schaffner, Stephen Ansolabehere, and Marissa Shih, "Cooperative Election Study Common Content, 2022," Harvard Dataverse, V2, available https://doi.org/10.7910/DVN/PR4L8P (last accessed May 11, 2024).

G Difference in Means

Table S7: Difference of Means Test-All Respondents

	Florida					North Carolina				
Group	NC-No cue	FL-Polarization	NC-Polarization	FL-Primary Cue	NC-Primary Cue	**NC No Cue**	FL-Polarization	NC-Polarization	FL-Primary Cue	NC-Primary Cue
FL-Polarization	.003	–	–	–	–	.002	–	–	–	–
NC-Polarization	.027	.030	–	–	–	.005	.007	–	–	–
FL-Primary Cue	.092***	.089***	.119***	–	–	.176***	.178***	.171***	–	–
NC-Primary Cue	.238***	.241***	.211***	.330***	–	.153***	.151***	.158***	.329***	–
**FL No Cue**	.006	.003	.032	.086***	.244***	.038	.040	.032	.138**	.268***

Notes: Table entries are unweighted difference in means for respondents who passed attention check. Conditions recoded where (1=NPA) (0=R, D, Minor Party). **FL No Cue** in **bold** in the row for the FL sample is the control group. **NC No Cue** in **bold** in the column for the NC sample is the control group.

Table S8: Difference of Means Test-Independents

	Florida					North Carolina				
Group	NC-No cue	FL-Polarization	NC-Polarization	FL-Primary Cue	NC-PrimaryCue	**NC-No cue**	FL-Polarization	NC-Polarization	FL-Primary Cue	NC-Primary Cue
FL-Polarization	.035	–	–	–	–	.068	–	–	–	–
NC-Polarization	.040	.075*	–	–	–	.053	.016	–	–	–
FL-Primary Cue	.173***	.139***	.213***	–	–	.337***	.269***	.284***	–	–
NC-Primary Cue	.160***	.195***	.120***	.334***	–	.082	.150**	.134**	.419***	–
**FL-No Cue**	.045	.010	.085*	.128***	.206***	.014	.082	.066	.351***	.068

Notes: Table entries are unweighted difference in means for respondents who passed attention check. Conditions recoded where (1=NPA) (0=R, D, Minor Party). **FL No Cue** in **bold** in the row for the FL sample is the control group. **NC No Cue** in **bold** in the column for the NC sample is the control group.

Table S9: Difference of Means Test-Partisans

	Florida					North Carolina				
Group	NC-No cue	FL-Polarization	NC-Polarization	FL-Primary Cue	NC-Primary Cue	**NC-No cue**	FL-Polarization	NC-Polarization	FL-Primary Cue	NC-Primary Cue
FL-Polarization	.011	–	–	–	–	.038	–	–	–	–
NC-Polarization	.003	.007	–	–	–	.088	.050	–	–	–
FL-Primary Cue	.032	.021	.029	–	–	.018	.055	.106	–	–
NC-Primary Cue	.288***	.289***	.291***	.320***	–	.206***	.168**	.118	.224***	–
**FL-No Cue**	.004	.015	.008	.036	.283***	.040	.077	.128*	.022	.245***

Notes: Table entries are unweighted difference in means for respondents who passed attention check. Conditions recoded where (1=NPA) (0=R, D, Minor Party). **FL No Cue** in **bold** in the row for the FL sample is the control group. **NC No Cue** in **bold** in the column for the NC sample is the control group.

H Principles and Guidance for Human Subjects Research

For the sake of anonymity, at this time we cannot disclose detailed information about the survey, but it was conducted by scholars at three southern universities. All three universities independently granted exempt IRB approval. Respondents in both the Florida and North Carolina surveys were randomly selected from the publicly available voter files from both states. There were no anticipated ethical issues associated with the administration of this survey, which asked standard (and unobtrusive) questions of human subjects. Based on the APSA Council's Principles and Guidance for Human Subjects Research document, we should note the following regarding human participants involved in our survey: (1) we sought voluntary and informed consent with written language preceding the start of the online survey, which identified the institution and principal investigators conducting the survey, explained that a participant can terminate the survey at any time, that a participant's identification is strictly confidential, and that the purpose of the survey was to conduct a research study to better understand the political opinions of voters living in the two states; (2) the survey did not involve any deception; (3) the survey did not intervene in political processes; and (4) the survey asked routine political and demographic questions of respondents and hence involved minimal risk. Additionally, we did not compensate (nor did we promise to compensate) respondents for their participation in our survey, which they were free to terminate at any point. We chose not to compensate participants because the survey took the typical respondent about 5 to 10 minutes to complete, and, undoubtedly because of their inherent interest in the subject.

I Pre-Registration

The Rise of Registered Independents in a Polarized America (AsPredicted #138356)

Created: 07/13/2023 10:46 AM (PT)

Author(s) [redacted for anonymity]

1) Have any data been collected for this study already?

No, no data have been collected for this study yet.

2) What's the main question being asked or hypothesis being tested in this study?

The main question we address is why there is a rise in registered independent voters ("No Party Affiliation" in Florida and "unaffiliated" in North Carolina) while the American public is becoming increasingly polarized and hence partisanship is actually increasing in strength (e.g., evident in ANES time series data on strength of partisanship). We test two sets of hypotheses using an experiment embedded in two surveys of registered voters in Florida and North Carolina. First, we test whether exposure to the institutional rules governing a state's party primary system affects how respondents think about party registration. State law varies regarding who may participate in a party primary. North Carolina allows unaffiliated voters to participate in major party primaries. After randomly receiving this information in a scenario, we expect respondents exposed to the information treatment will be more likely to advise a friend moving to NC to register as an unaffiliated voter, since their friend may vote in either major party's primary election. By comparison, we expect respondents exposed to the party primary rules treatment in Florida, which has a closed primary system will be more likely to advise their friend to register with the party with which they are registered or that aligns with their party identification (PID) self-placement. The control is providing no information about party registration in FL (or NC).

Second, we test whether exposure to the polarized political climate in a state affects how respondents think about party registration. We randomly assign respondents to receive the following scenario: "A friend of yours is moving to Florida (North Carolina). They are interested in registering to vote. As you may know, as in North Carolina (Florida), Democratic and Republican politicians are constantly fighting over hot-button issues in Florida (North Carolina). This has been the state of affairs in Florida (North Carolina) for decades now, and likely will not change anytime soon." We expect respondents exposed to this treatment will be more likely to advise their friend to register as an independent (no party affiliation in FL / unaffiliated in NC).

3) Describe the key dependent variable(s) specifying how they will be measured.

The dependent variable-political party registration-is measured as a categorical variable for Democrat, Republican, Independent, Third party, not registered. We ask survey respondents to identify the party with which they advise a friend to register.

4) How many and which conditions will participants be assigned to?

Participants will be assigned to one of 6 conditions (including a pure control) in a 2 (factual information on party primary rules: FL/NC) x 2 (hyper-polarized state: polarized / non polarized: FL/NC) between-subjects design. We will assign participants evenly across conditions (i.e., 1,000 participants per condition).

5) Specify exactly which analyses you will conduct to examine the main question/hypothesis.

Compute descriptive statistics (M; SD; N) for the outcome measure. Compare means across conditions. We will not correct for multiple comparisons but we will instead report all comparisons (Rothman, 1990; Saville, 1990). Regress the outcome variable on the experimental conditions. We will run these regressions without controls. We will use OLS regressions with robust Huber-White standard errors for the continuous outcome measure. Our baseline category will be participants who were assigned to the control group. We will not include demographics in our main models. However, we will add them in robustness checks. We will use two-tailed tests of significance.

6) Describe exactly how outliers will be defined and handled, and your precise rule(s) for excluding observations.

In the main text, we will report the results only for participants who passed the manipulation checks. In the appendix, we will also report the results for participants who failed the manipulation checks.

7) How many observations will be collected or what will determine sample size? No need to justify decision, but be precise about exactly how the number will be determined.

1,000 observations per cell, for a total of 6,000 participants give the desired power = 0.8 and beta = 0.05 with a Cohen's d $\tilde{0}$.23.

8) Anything else you would like to pre-register? (e.g., secondary analyses, variables collected for exploratory purposes, unusual analyses planned?)

The survey of registered Florida and North Carolina voters includes other variables, which we will assess for descriptive analyses and controls, including: party registration, political participation, past vote history in primary elections, party identification (7-point scale), political interest, self-monitor scale, ide-

ology (7-point scale), prior party registration, and standard demographics.

J Questionnaire

Questionnaire

Are you currently a resident of Florida [North Carolina]?

- Yes (1)
- No (2)
- Don't know (9)

Are you currently registered to vote in Florida [North Carolina]?

- Yes (1)
- No (2)
- Don't know (9)

During the past year did you ...

	Yes (1)	No (2)	Don't know (9)
Attend local political meetings (such as school board or city council) (1)	○	○	○
Put up a political sign (such as a lawn sign or bumper sticker) (2)	○	○	○
Work for a candidate or campaign (3)	○	○	○
Attend a political protest, march or demonstration (4)	○	○	○
Contact a public official (5)	○	○	○
Donate money to a candidate, campaign, or political organization (6)	○	○	○

Some people believe that voting in primary elections is very important and others believe it is not important at all. In your opinion, voting in primary elections is...

- Very important (1)
- Important (2)
- Not important (3)
- Not important at all (4)
- Don't know (9)

Generally speaking, do you usually think of yourself as a Republican, a Democrat, or an Independent?

- Republican (1)
- Democrat (2)
- Independent (3)
- Other (4)
- Don't know (5)

Would you call yourself a strong Republican or a not very strong Republican?

- Strong Republican (1)
- Not very strong Republican (2)
- Don't know (3)

Would you call yourself a strong Democrat or a not very strong Democrat?

- Strong Democrat (1)
- Not very strong Democrat (2)
- Don't know (3)

Do you think of yourself as closer to the Republican Party or the Democratic Party?

- Closer to the Republican Party (1)
- Closer to the Democratic Party (2)
- Neither (3)

Some people follow what's going on in government and public affairs most of the time, whether there's an election going on or not. Others aren't that interested. Would you say you follow what's going on in government and public affairs ...

- Most of the time (1)
- Some of the time (2)
- Only now and then (3)
- Hardly at all (4)
- Don't know (9)

Do you agree or disagree with this statement: I prefer to surround myself with politically like minded people.

- Agree (1)
- Disagree (2)
- Don't know (3)

Attention Check To ensure you are a real person, please select the color red.

- Red (1)
- White (2)
- Blue (3)

Condition1 Now we'd like you to consider the following scenario:

A friend of yours is moving to [respondent's home state] Florida/North Carolina. They are interested in registering to vote.

How would you advise your friend to register to vote in [respondent's home state] Florida/North Carolina?

- Register with the Democratic Party (1)
- Register with the Republican Party (2)
- Register as Unaffiliated (no party affiliation) (3)
- Register with a third party (4)
- Don't know (9)

Treatment1 Now we'd like you to consider the following scenario:

A friend of yours is moving to [not respondent's home state] Florida/North Carolina. They are interested in registering to vote.

How would you advise your friend to register to vote in [not respondent's home state] Florida/North Carolina?

- Register with the Democratic Party (1)
- Register with the Republican Party (2)
- Register as Unaffiliated (no party affiliation) (3)
- Register with a third party (4)
- Don't know (9)

Treatment2 Now we'd like you to consider the following scenario:

A friend of yours is moving to Florida. They are interested in registering to vote. As you may know, as in North Carolina, Democratic and Republican politicians in Florida are constantly fighting over

hot-button issues. This has been the state of affairs in Florida for decades now, and likely will not change anytime soon.

How would you advise your friend to register to vote in Florida?

- Register with the Democratic Party (1)
- Register with the Republican Party (2)
- Register as Unaffiliated (no party affiliation) (3)
- Register with a third party (4)
- Don't know (9)

Treatment3 Now we'd like you to consider the following scenario:

A friend of yours is moving to North Carolina. They are interested in registering to vote. As you may know, Democratic and Republican politicians are constantly fighting over hot-button issues in North Carolina. This has been the state of affairs in North Carolina for decades now, and likely will not change anytime soon.

How would you advise your friend to register to vote in North Carolina?

- Register with the Democratic Party (1)
- Register with the Republican Party (2)
- Register as Unaffiliated (no party affiliation) (3)
- Register with a third party (4)
- Don't know (9)

Treatment4 Now we'd like you to consider the following scenario:

A friend of yours is moving to Florida. They are interested in registering to vote. As you may know, in the state of Florida, registered Independents cannot participate in either major party's (Democratic or Republican) primary election. How would you advise your friend to register to vote in Florida?

- Register with the Democratic Party (1)
- Register with the Republican Party (2)
- Register as Unaffiliated (no party affiliation) (3)
- Register with a third party (4)
- Don't know (9)

Treatment5 Now we'd like you to consider the following scenario:

A friend of yours is moving to North Carolina. They are interested in registering to vote. As you may know, in the state of North Carolina, registered Independents can participate in either major party's (Democratic or Republican) primary election.

How would you advise your friend to register to vote in North Carolina?

- Register with the Democratic Party (1)
- Register with the Republican Party (2)
- Register as Unaffiliated (no party affiliation) (3)
- Register with a third party (4)
- Don't know (9)

Manipulation Check In the example you just read, who was the person who was moving?

- A colleague (1)
- A parent (2)
- A friend (3)

We hear a lot of talk these days about liberals and conservatives. Here is a seven-point scale on which the political views that people might hold are arranged from extremely liberal to extremely conservative. Where would you place yourself on this scale?

- Very liberal (1)
- Liberal (2)
- Slightly liberal (3)
- Moderate (4)
- Slightly conservative (5)
- Conservative (6)
- Very conservative (7)

Did you move to Florida from another state?

- Yes (1)
- No (2)
- Don't know/Prefer not to answer (9)

In what year were you born?

▼ 2005 (2005) ... 1923 or before (1923)

What is your gender?

- Male (1)
- Female (2)
- Other (3)

What racial or ethnic group best describes you?

- White (1)
- Black or African American (2)
- Hispanic or Latino (3)
- Asian or Asian American (4)
- Native American (5)
- Middle Eastern (6)
- Two or more races (7)
- Other (list) (8) ____

Are you of Latino, Hispanic, or Spanish origin or descent?

- No (1)
- Yes (2)

What is the highest level of education you have achieved?

- Less than a High School Degree (1)
- High School Graduate or equivalent (ex: GED) (2)
- Some College (including Associate Degree) (3)
- College Graduate (Bachelor’s Degree) (4)
- Some Graduate Work, did not receive advanced degree (5)
- Graduate Degree (6)
