# Supplementary figures and images for: Treating a friend to voter registration in a Divided America
Source: PLoS One. 2025 Dec 16;20(12):e0337176. doi: 10.1371/journal.pone.0337176 (PMC12707647; doi:10.1371/journal.pone.0337176)

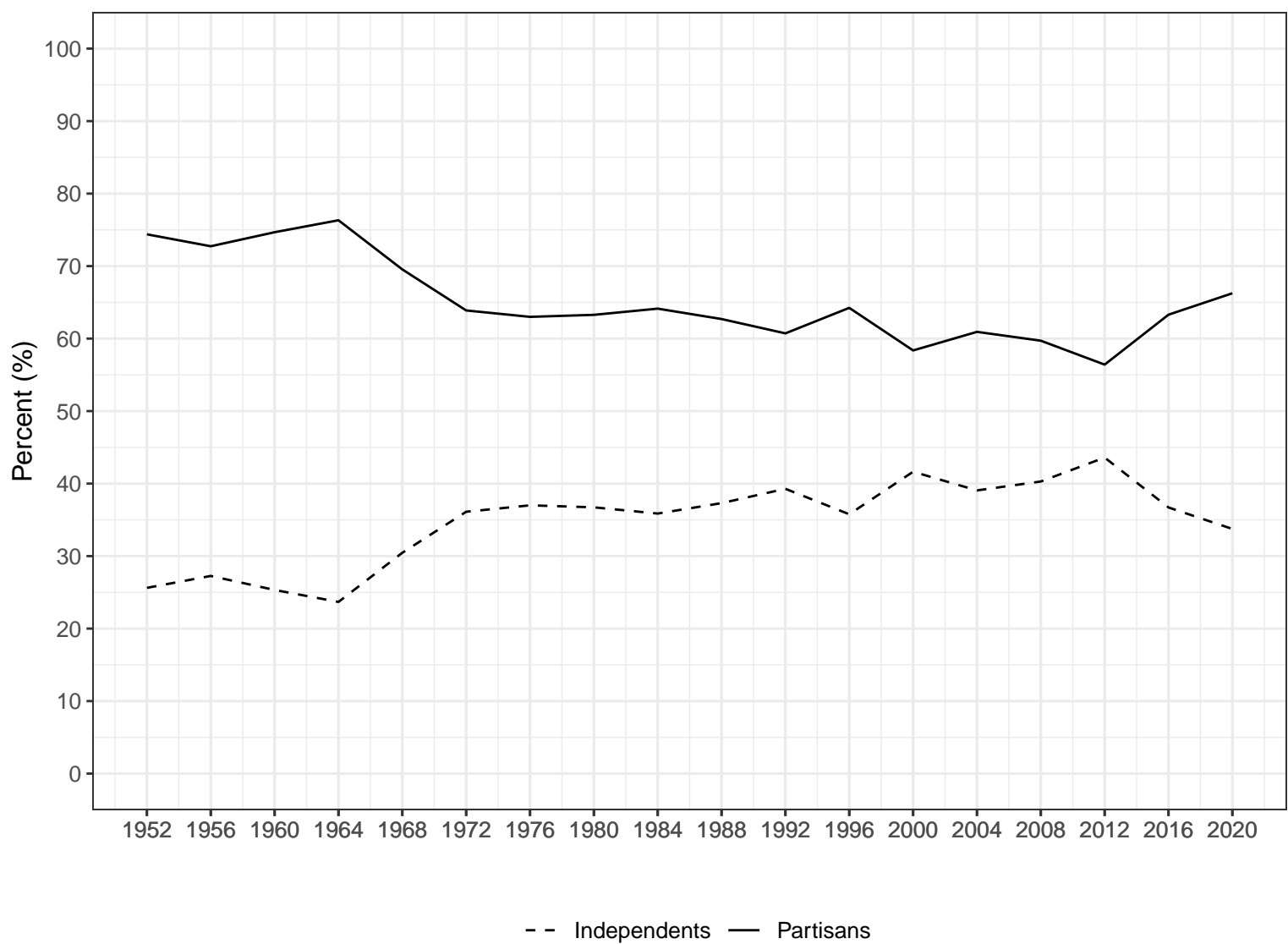

Supplement: S1 Fig — (PDF) [file pone.0337176.s008.pdf]

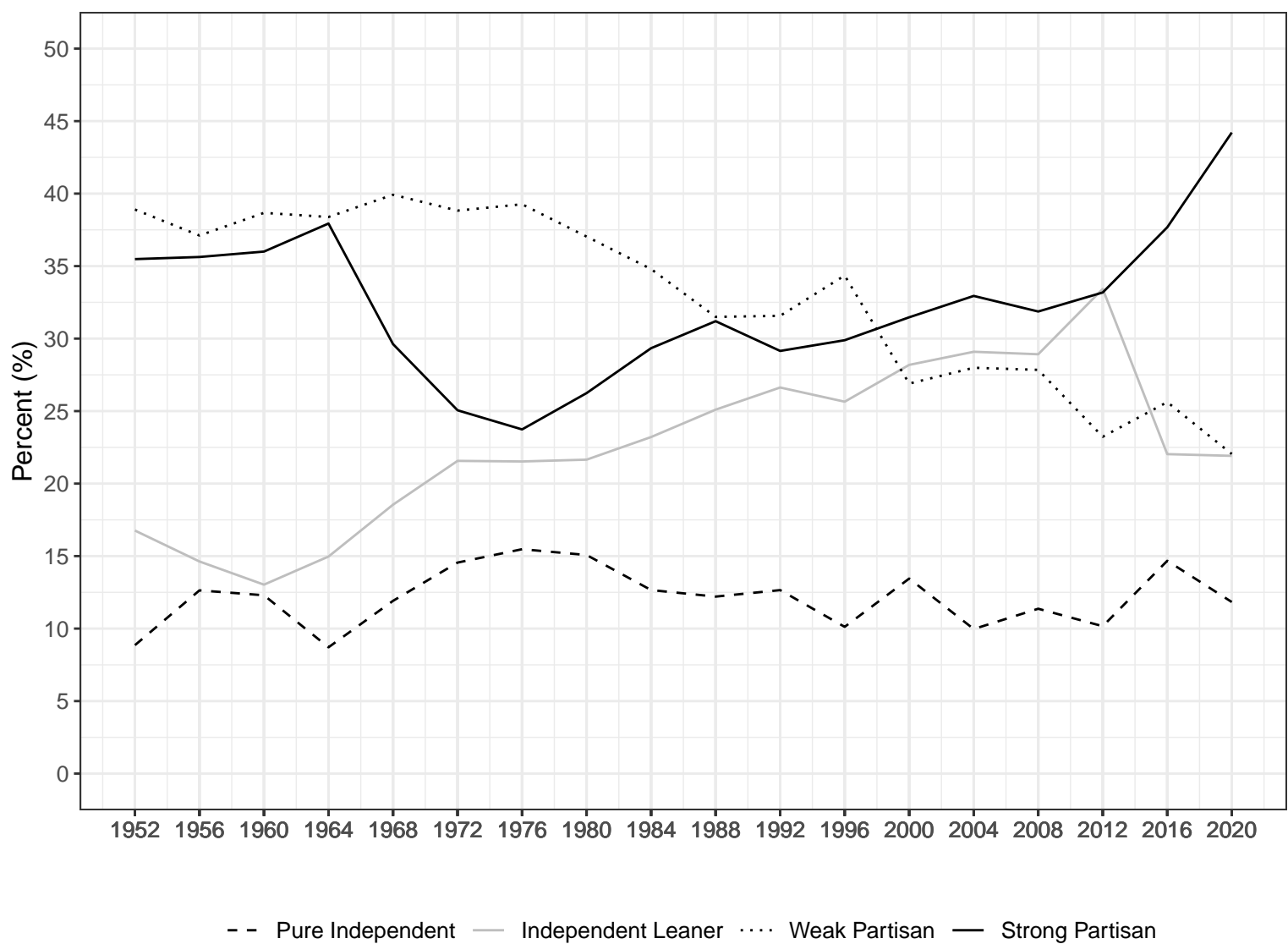

Supplement: S2 Fig — (PDF) [file pone.0337176.s009.pdf]

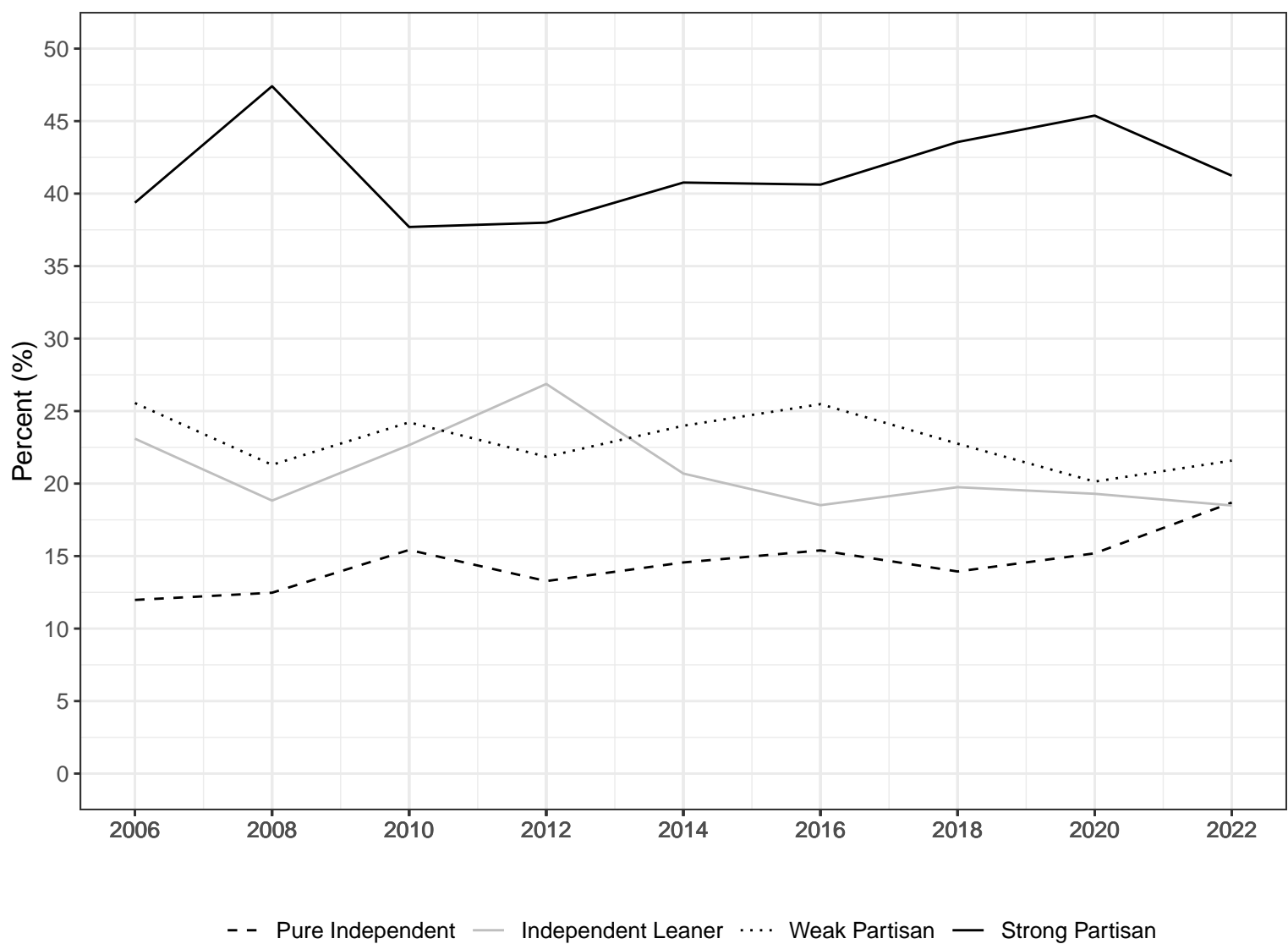

Supplement: S3 Fig — (PDF) [file pone.0337176.s010.pdf]
