# Supplementary material for: Treating a friend to voter registration in a Divided America
Source: PLoS One. 2025 Dec 16;20(12):e0337176. doi: 10.1371/journal.pone.0337176 (PMC12707647; doi:10.1371/journal.pone.0337176)
Supplement: S1 Text — (PDF) [file pone.0337176.s014.pdf]

Questionnaire

Are you currently a resident of Florida [North Carolina]?

- Yes (1)
- No (2)
- Don't know (9)

Are you currently registered to vote in Florida [North Carolina]?

- Yes (1)
- No (2)
- Don't know (9)

During the past year did you ...

	Yes (1)	No (2)	Don't know (9)
Attend local political meetings (such as school board or city council) (1)	○	○	○
Put up a political sign (such as a lawn sign or bumper sticker) (2)	○	○	○
Work for a candidate or campaign (3)	○	○	○
Attend a political protest, march or demonstration (4)	○	○	○
Contact a public official (5)	○	○	○
Donate money to a candidate, campaign, or political organization (6)	○	○	○

Some people believe that voting in primary elections is very important and others believe it is not important at all. In your opinion, voting in primary elections is...

- Very important (1)
- Important (2)
- Not important (3)
- Not important at all (4)
- Don't know (9)

Generally speaking, do you usually think of yourself as a Republican, a Democrat, or an Independent?

- Republican (1)
- Democrat (2)
- Independent (3)
- Other (4)
- Don't know (5)

Would you call yourself a strong Republican or a not very strong Republican?

- Strong Republican (1)
- Not very strong Republican (2)
- Don't know (3)

Would you call yourself a strong Democrat or a not very strong Democrat?

- Strong Democrat (1)
- Not very strong Democrat (2)
- Don't know (3)

Do you think of yourself as closer to the Republican Party or the Democratic Party?

- Closer to the Republican Party (1)
- Closer to the Democratic Party (2)
- Neither (3)

Some people follow what's going on in government and public affairs most of the time, whether there's an election going on or not. Others aren't that interested. Would you say you follow what's going on in government and public affairs ...

- Most of the time (1)
- Some of the time (2)
- Only now and then (3)
- Hardly at all (4)
- Don't know (9)

Do you agree or disagree with this statement: I prefer to surround myself with politically like minded people.

- Agree (1)
- Disagree (2)
- Don’t know (3)

Attention Check To ensure you are a real person, please select the color red.

- Red (1)
- White (2)
- Blue (3)

Condition1 Now we’d like you to consider the following scenario:

A friend of yours is moving to [respondent’s home state] Florida/North Carolina. They are interested in registering to vote.

How would you advise your friend to register to vote in [respondent’s home state] Florida/North Carolina?

- Register with the Democratic Party (1)
- Register with the Republican Party (2)
- Register as Unaffiliated (no party affiliation) (3)
- Register with a third party (4)
- Don't know (9)

Treatment1 Now we’d like you to consider the following scenario:

A friend of yours is moving to [not respondent’s home state] Florida/North Carolina. They are interested in registering to vote.

How would you advise your friend to register to vote in [not respondent’s home state] Florida/North Carolina?

- Register with the Democratic Party (1)
- Register with the Republican Party (2)
- Register as Unaffiliated (no party affiliation) (3)
- Register with a third party (4)
- Don't know (9)

Treatment2 Now we’d like you to consider the following scenario:

A friend of yours is moving to Florida. They are interested in registering to vote. As you may know, as in North Carolina, Democratic and Republican politicians in Florida are constantly fighting over

hot-button issues. This has been the state of affairs in Florida for decades now, and likely will not change anytime soon.

How would you advise your friend to register to vote in Florida?

- Register with the Democratic Party (1)
- Register with the Republican Party (2)
- Register as Unaffiliated (no party affiliation) (3)
- Register with a third party (4)
- Don't know (9)

Treatment3 Now we'd like you to consider the following scenario:

A friend of yours is moving to North Carolina. They are interested in registering to vote. As you may know, Democratic and Republican politicians are constantly fighting over hot-button issues in North Carolina. This has been the state of affairs in North Carolina for decades now, and likely will not change anytime soon.

How would you advise your friend to register to vote in North Carolina?

- Register with the Democratic Party (1)
- Register with the Republican Party (2)
- Register as Unaffiliated (no party affiliation) (3)
- Register with a third party (4)
- Don't know (9)

Treatment4 Now we'd like you to consider the following scenario:

A friend of yours is moving to Florida. They are interested in registering to vote. As you may know, in the state of Florida, registered Independents cannot participate in either major party's (Democratic or Republican) primary election. How would you advise your friend to register to vote in Florida?

- Register with the Democratic Party (1)
- Register with the Republican Party (2)
- Register as Unaffiliated (no party affiliation) (3)
- Register with a third party (4)
- Don't know (9)

Treatment5 Now we'd like you to consider the following scenario:

A friend of yours is moving to North Carolina. They are interested in registering to vote. As you may know, in the state of North Carolina, registered Independents can participate in either major party's (Democratic or Republican) primary election.

How would you advise your friend to register to vote in North Carolina?

- Register with the Democratic Party (1)
- Register with the Republican Party (2)
- Register as Unaffiliated (no party affiliation) (3)
- Register with a third party (4)
- Don't know (9)

Manipulation Check In the example you just read, who was the person who was moving?

- A colleague (1)
- A parent (2)
- A friend (3)

We hear a lot of talk these days about liberals and conservatives. Here is a seven-point scale on which the political views that people might hold are arranged from extremely liberal to extremely conservative. Where would you place yourself on this scale?

- Very liberal (1)
- Liberal (2)
- Slightly liberal (3)
- Moderate (4)
- Slightly conservative (5)
- Conservative (6)
- Very conservative (7)

Did you move to Florida from another state?

- Yes (1)
- No (2)
- Don't know/Prefer not to answer (9)

In what year were you born?

▼ 2005 (2005) ... 1923 or before (1923)

What is your gender?

- Male (1)
- Female (2)
- Other (3)

What racial or ethnic group best describes you?

- White (1)
- Black or African American (2)
- Hispanic or Latino (3)
- Asian or Asian American (4)
- Native American (5)
- Middle Eastern (6)
- Two or more races (7)
- Other (list) (8) ____

Are you of Latino, Hispanic, or Spanish origin or descent?

- No (1)
- Yes (2)

What is the highest level of education you have achieved?

- Less than a High School Degree (1)
- High School Graduate or equivalent (ex: GED) (2)
- Some College (including Associate Degree) (3)
- College Graduate (Bachelor’s Degree) (4)
- Some Graduate Work, did not receive advanced degree (5)
- Graduate Degree (6)
